# Molecular Sub-Classification of Renal Epithelial Tumors Using Meta-Analysis of Gene Expression Microarrays

**DOI:** 10.1371/journal.pone.0021260

**Published:** 2011-07-27

**Authors:** Thomas Sanford, Paul H. Chung, Ariel Reinish, Vladimir Valera, Ramaprasad Srinivasan, W. Marston Linehan, Gennady Bratslavsky

**Affiliations:** 1 Urologic Oncology Branch, National Cancer Institute, Bethesda, Maryland, United States of America; 2 Department of Urology, Upstate Medical University, State University of New York, Syracuse, New York, United States of America; Baylor College of Medicine, United States of America

## Abstract

**Purpose:**

To evaluate the accuracy of the sub-classification of renal cortical neoplasms using molecular signatures.

**Experimental Design:**

A search of publicly available databases was performed to identify microarray datasets with multiple histologic sub-types of renal cortical neoplasms. Meta-analytic techniques were utilized to identify differentially expressed genes for each histologic subtype. The lists of genes obtained from the meta-analysis were used to create predictive signatures through the use of a pair-based method. These signatures were organized into an algorithm to sub-classify renal neoplasms. The use of these signatures according to our algorithm was validated on several independent datasets.

**Results:**

We identified three Gene Expression Omnibus datasets that fit our criteria to develop a training set. All of the datasets in our study utilized the Affymetrix platform. The final training dataset included 149 samples represented by the four most common histologic subtypes of renal cortical neoplasms: 69 clear cell, 41 papillary, 16 chromophobe, and 23 oncocytomas. When validation of our signatures was performed on external datasets, we were able to correctly classify 68 of the 72 samples (94%). The correct classification by subtype was 19/20 (95%) for clear cell, 14/14 (100%) for papillary, 17/19 (89%) for chromophobe, 18/19 (95%) for oncocytomas.

**Conclusions:**

Through the use of meta-analytic techniques, we were able to create an algorithm that sub-classified renal neoplasms on a molecular level with 94% accuracy across multiple independent datasets. This algorithm may aid in selecting molecular therapies and may improve the accuracy of subtyping of renal cortical tumors.

## Introduction

Renal epithelial tumors are a diverse group of neoplasms that have been sub-classified based on histologic morphology [1]. The four most common types of renal cortical neoplasms are clear cell renal cell carcinoma (RCC) (63–89%,), papillary RCC (7–19%), chromophobe RCC (2–6%), and oncocytoma (5–7%) [2], [3]. Clear cell tumors are most commonly associated with mutations in the *VHL* tumor suppressor gene, familial and a subset of papillary (type I) tumors are associated with dysregulation of the *MET* proto-oncogene, and familial chromophobe tumors and oncocytomas are associated with dysregulation of the *BHD* gene [4], [5], [6], [7], [8].

Improved understanding of the genetic alterations and downstream molecular pathways of the histologic subtypes of renal epithelial neoplasms has led to the development of targeted molecular therapies and the tailoring of treatment and follow-up to the subtype of the tumor. Knowledge of the aggressiveness of histologic subtypes has aided in determining which patients may be candidates for surveillance, as some non-clear cell subtypes are associated with a more indolent course [3]. The FDA has approved a number of targeted therapies for clear cell histology and there are now promising clinical trials underway for papillary histology [9], [10].

The ability of sub-classification to aide in prognostication and selection of appropriate treatment emphasizes the importance of accuracy in the sub-typing of renal cortical tumors. Unfortunately, diagnostic concordance between pathologists may be suboptimal. This has recently been demonstrated by Kummerlin et al, who showed that pathologists disagreed on the sub-classification of non-clear cell tumors in up to 50% of cases [11]. While immunohistochemistry is a valuable adjunct, most markers lack either specificity or sensitivity, and even combinations of markers achieve only 78–86% agreement with morphology-based sub-classification [12].

In the current study, we used meta-analysis of gene expression microarray data in an attempt to provide a link between histopathologic diagnosis and molecular characteristics. By incorporating data from multiple institutions, we aimed to generate a large enough dataset to create a highly genereralizeable set of signatures that represent the molecular correlate of the four major sub-types of renal epithelial tumors.

## Materials and Methods

### Dataset search

The Gene Expression Omnibus (GEO) and Array Express databases were searched for published microarray datasets involving renal neoplasms. Search terms were: “kidney tumors,” “kidney cancer,” and “renal cell carcinoma.” Selected datasets included the four major types of renal cortical neoplasms: clear cell, papillary, chromophobe, oncocytoma.

We included only datasets that utilized the Affymetrix platform because this platform has demonstrated the best inter-institution agreeability [13]. All studies processed samples similarly according to the Affymetrix protocol. Datasets without a reference to a publication describing the source of their tissue and IRB approval were excluded. All studies without feature-level extraction output files (.CEL files) were excluded in an attempt to prevent confounding effects of different algorithms used in data pre-processing [14]. Samples within each dataset with a histologic sub-classification other than the four most common types of renal epithelial tumors listed above were also excluded. If a sample was noted to have features of another histology (i.e. clear cell with papillary features), the sample was excluded. All the datasets included in our training and validation samples were comprised of snap-frozen samples from surgically removed primary tumors; none of the studies used tissue from biopsies and none of the samples were derived from metastatic sites.

### Normalization and Filtering

Raw data from each dataset were imported into BRB array tools (http://linus.nci.nih.gov/BRB-ArrayTools.html). Each dataset was normalized independently using the GC-RMA algorithm [15], [16]. Each dataset was filtered so that genes were excluded when less than 20% of expression data had at least a 1.5 -fold change in either direction from gene's median value, and when the percent of data missing or filtered out exceeded 50%. Genes passing the filtering criteria from each dataset were intersected using the MergeMaid package for the R environment (http://www.r-project.org) to identify common genes across multiple datasets. Next, the “IntCor” function of MergeMaid was used to perform integrative correlation analysis, whereby the correlation of expression values across samples within one dataset, and across multiple datasets, was evaluated [17]. All genes with an integrative correlation coefficient of less than 0.5 were excluded. Thus, the final list of genes included in our analysis represented the genes with the least amount of variability across multiple datasets.

### Algorithm Construction and Meta-Analysis

Multi-dimensional scaling was performed to determine the differences in gene expression between datasets. Unsupervised hierarchical clustering analysis was then performed on each dataset independently to look for clustering patterns consistent across multiple datasets. To maximize the differences between the classes, and thus augment our predictive power, we sought to create signatures that could be applied in an algorithm that would mimic the natural clustering of samples in unsupervised analysis. The creation of predictive signatures consisted of two steps as previously described by Dobbin et al: 1) Differentially expressed genes between classes were identified 2) The genes with the highest discriminatory ability were selected from the differentially expressed genes [18]. To identify differentially expressed genes across multiple datasets, we employed a non-parametric ‘rank product’ method implemented in the RankProd package for the R environment [19]. This method has been shown to have higher sensitivity and specificity than other types of meta-analytic tools for microarrays [20]. Class comparison analysis using RankProd identified differentially expressed genes between two classes in each signature in the algorithm. We pre-specified a significant p-value and pfp (“percent false prediction” - a measure of false discovery rate) as less than 0.001. Once differentially expressed genes were identified, feature selection was performed using a pair-based pairs method in BRB-array tools termed “greedy pairs” [15], [21]. We set the number of pairs at 25 for each signature, resulting in 50 gene signatures. We then decreased the number of pairs to 12 (24 gene signatures) and then to 5 (10 gene signatures) to determine the effect of decreasing the size of the signature to on the accuracy of sub-classification.

### Validation

We began validating our signatures by performing k-fold cross-validation on the training set using the tools provided by BRB-array tools [15]. We then searched the Gene Expression Omnibus and Array Express databases using the same search criteria as before to identify independent datasets that could be used for validation. We included all datasets that met our criteria and that were not used in the training set and again excluded all samples without an associated reference describing the source of tissue and IRB approval. We also validated our signatures on one of our own datasets from a prior IRB-approved study [22]. Finally, we applied our signatures in a sequential fashion according to our algorithm and used the nearest centroid classification and the Bayesian compound covariate predictors to evaluate the efficacy of the signatures in our algorithm [15], [23], [24]. The error rate was determined by misclassification at any step during the algorithm.

## Results

### Training set

A search of public databases identified four datasets that utilized the Affymetrix platform and included samples with the four major subtypes of renal epithelial tumors. The associated GEO accession numbers are as follows: GSE 15641, GSE11024, GSE11151, and GSE2109. One dataset, GSE2109 was excluded because it was not associated with a peer-reviewed publication describing the source of the samples. A summary of the three remaining datasets are shown in Table 1 [25], [26], [27].

**Table 1:** Summary of datasets included in training set.

GEO ID	GSE15641	GSE11024	GSE11151	
PubMed ID*	16115910	18519660	19445733	
Institution	Beth Israel	Van Andel	Heidelberg	
Chip type	HG U133A	HG U133_Plus_2.0	HG U133_Plus_2.0	Total
Clear cell	32	11	27	70
Papillary	11	10	19	40
Chromophobe	6	6	4	16
Oncocytoma	12	7	4	23
Total	61	34	54	149

*Pubmed ID of associated reference.

### Normalization and Filtering

After normalization, 10,092, 19,601, and 23,870 genes passed filtering criteria for GSE 15641, GSE11024, GSE11151, respectively. Intersection of the genes using probe set IDs revealed 8,247 genes common to all three datasets after filtering. Integrative correlation analysis identified 2,932 genes with an integrative correlation coefficient less than 0.5. These genes were discarded, leaving 5,315 genes to be used in the creation of our signatures.

### Multi-Dimensional Scaling

**Figure 1:**
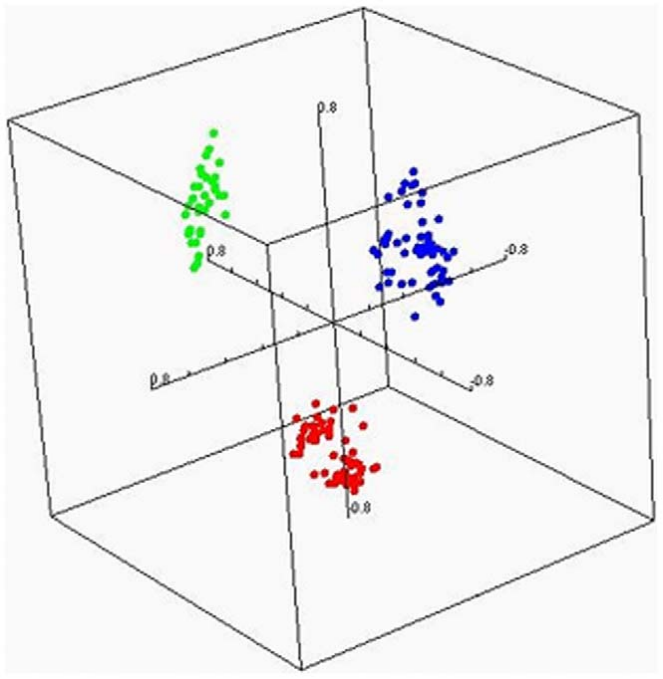
Multi-dimensional scaling of gene expression profiles from samples from different institutions. Red: GSE 11024, Green: GSE 11151, Blue GSE 15641.

Multi-dimensional scaling of samples revealed differences in average gene expression values between datasets (Figure 1). The differences between datasets indicated the need for statistical methods that take the different sources of the data into account.

### Algorithm Creation

**Figure 2:**
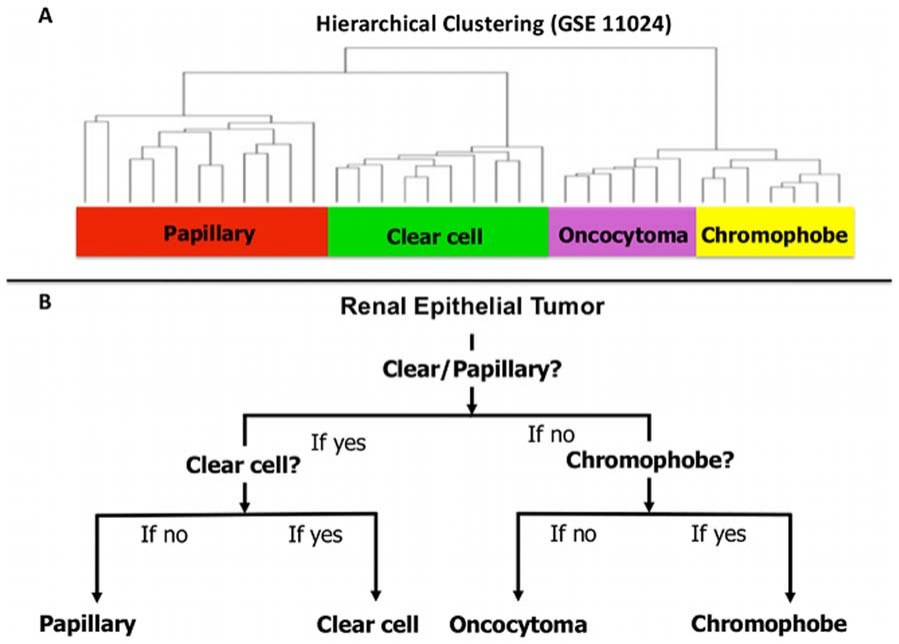
Clustering of renal epithelial tumors. A) Natural clustering pattern of renal epithelial tumors, demonstrated with GSE11024. B) Algorithm used to sub-classify renal epithelial tumors according to their molecular characteristics closely mimics the natural clustering pattern.

Unsupervised clustering was performed independently for each dataset and revealed consistent trends, as is illustrated in Figure 2A. The first division separated datasets into samples of clear/papillary and chromophobe/oncocytoma. Within these two primary divisions, samples were separated into clusters of clear cell, papillary, chromophobe, and oncocytoma at intermediate branch points. Knowledge of clustering was used in creating the algorithm; we applied our signatures at the branch points observed in the natural clustering pattern in order to maximize the differences between our samples. The algorithm (Figure 2B) consisted of three signatures. The algorithm was applied to samples in a two-step process using the first signature to separate samples into clear cell/papillary or chromophobe/oncocytoma. One of the two remaining signatures was then applied to the sample depending on the results of the first signature, which separated the sample into one of the four sub-types of renal epithelial neoplasms.

### Meta-Analysis and Signature Creation

Class comparison analysis was performed to identify differentially expressed genes for each signature. Thus, three separate class comparison analysis comparison analyses were performed using the RankProd package. Class comparison analysis between clear/papillary and chromophobe/oncocytoma identified 2,008 differentially expressed genes. There were 1,408 differentially expressed genes in the clear cell vs. papillary class comparison, and 432 differentially expressed genes in the chromphobe vs. oncocytoma class comparison. The greedy pairs feature selection process chose the most predictive genes among each list of differentially expressed genes to create our final signatures.

### Validation

K-fold cross-validation using the nearest centroid classifier in BRB-array tools resulted in a correct classification rate of 99% for the clear cell/papillary versus chromophobe/oncocytoma comparison, 98% for the clear cell vs papillary comparison, and 95% for the chromophobe vs oncocytoma comparison. Search of the Gene Expression Omnibus database for independent validation datasets identified 4 independent datasets from three separate institutions that met our search criteria [28], [29], [30], [31]. We also used ten of our own clear cell samples from patients with known VHL mutations [22]. None of the samples in the validation datasets were part of the original training set. We first applied our 50 gene signatures as shown in Figure 2B using the nearest centroid classification predictor, and the correct classification rate was recorded. A summary of the results of correct classification based on our algorithm are shown in Table 2. Sixty-eight of the 72 samples were correctly classified. Of note, 49 of the total 52 (94%) of the non-clear cell histologies were correctly classified. The size of the signatures was decreased to 24 and then to 10 by decreasing the number of pairs of genes in the “greedy-pairs” function. As shown in Table 3, decreasing the number of genes in each signature did not affect the misclassification rate during validation. The results of the Bayesian compound covariate predictor are also shown in Table 3. The percent of incorrectly classified samples was lower with the Bayesian compound covariate predictor than with the nearest centroid, but when the un-classified samples were added, the two methods had similar results. Similar to the nearest centroid predictor, the Bayesian compound covariate model maintained discriminatory ability as the number of genes decreases. The full composition of these signatures may be found in Table S1.

**Table 2:** Summary of validation set and results of classification algorithm.

GEO ID#	GSE8271	GSE12090	GSE7023	GSE6344	—		
Institution	V.A.I.	Cornell	V.A.I.	M.C.	N.C.I.		
Pubmed ID*	18773095	17145811	17409424	17699851			
Chip type	HG U133_Plus_2.0	HG U133_Plus_2.0	HG U133_Plus_2.0	HG U133A	HG U133_Plus_2.0	Total	Correct Classification (%)
Clear Cell	—	—	—	10	10	20	19/20 (95%)
Papillary	—	—	14	—	—	14	14/14 (100%)
Chromophobe	10	9	—	—	—	19	17/19 (90%)
Oncocytoma	10	9	—	—	—	19	18/19 (95%)
Total	20	18	14	10	10	72	68/72 (94%)

*Publication associated with dataset.

V.A. – Van Andle Institute.

M.C. – Mayo Clinic, Jacksonville.

N.C.I. – National Cancer Institute.

**Table 3:** Results of validation with multiple methods and multiple signature sizes.

	NC* Training	NC* Validation	BCCP** Training	BCCP** Validation
Misclassification 50 genes	2.7%	5.6%	2.0%	4.2%
Misclassification 24 genes	1.3%	6.9%	2.0%	4.2%
Misclassification 10 genes	2.7%	5.6%	1.3%	5.6%
Non-classified 50 genes	—	—	0.7%	1.3%
Non-classified 24 genes	—	—	2.0%	2.7%
Non-classified 10 genes	—	—	6.0%	4.2%

*NC – Nearest Centroid.

**BCCP – Bayesian Compound Covariate Predictor.

## Discussion

Until reliable biomarkers and molecular prognostic tools gain widespread acceptance in the evaluation of renal masses, histopathologic diagnosis remains an important factor in prognostication, selection of treatment options, and inclusion in clinical trials. Unfortunately, pathologists often disagree on sub-classification of renal neoplasms based on morphology alone, with discordance rates as high as 50% in the non-clear-cell histologies [11]. This leads to reliance on immunohistochemistry, but immunohistochemistry and morphology are often at odds [12]. In this study, we attempted to improve the molecular correlate to morphology by developing gene expression signatures to aid in the classification of the most common types of renal cortical neoplasms.

Although sub-classification of renal neoplasms by molecular signatures has been described previously, the methods used lacked extensive external validation and were not amenable to being translated into clinically useful tests [28], [32]. In a study by Yang et al., diagnosis of 16 samples was based on hierarchical clustering among samples from prior studies. Furge et al. split a sample of cDNA arrays into two groups: a training set and a validation set. A 1018-set signature was used to predict the histology of their validation set through the use of a nearest shrunken centroid classifier [28]. While these studies demonstrate the feasibility of molecular signatures in the sub-classification of renal tumors, molecular signatures need to be reasonably sized and they need to undergo rigorous external validation before they are clinically useful [33].

A search of published datasets affords the opportunity to build large datasets, but the differences in gene expression values between datasets must be addressed. We limited our inter-dataset variation by excluding all studies that did not utilize the Affymetrix platform. Nevertheless, multi-dimensional scaling of the samples in our study revealed obvious differences in gene expression values between studies. Thus, we were obligated to ensure our statistical analysis considered the differences between datasets in addition to the differences between tumor types.

We took several measures to reduce the effects of inter-study variation. First, we performed an integration correlation analysis to discard all the genes with poor correlation and high variability within a given study and across studies. Second, when identifying differentially expressed genes to be included in our classifier, we used meta-analytic techniques that took the differences between datasets into account. This methodology may have the advantage of identifying the genes that are most consistently associated with the various histologies across multiple datasets, thus increasing the generalizability of the signatures in our algorithm. Finally, to maximize our power to distinguish between classes, we exploited the differences inherent in the biology of these tumors by utilizing knowledge of their natural clustering pattern. Interestingly, the initial branching observed in unsupervised clustering of all three datasets revealed clustering of clear and papillary RCC separate from chromophobe RCC and oncocytomas. This clustering pattern may be explained by the site of origin of these tumors. Chromophobe tumors and oncocytomas are derived from cells in the distal tubule whereas clear cell and papillary tumors are thought to arise from cells in the proximal tubule [34]. Within each sub-branch, the tumors further differentiate based on their histologic family. Therefore, while creating an algorithm, we sought to maximize the differences between samples while minimizing variance by applying our signatures at the first and second branch points.

We validated our signatures using the most stringent methods available to us [35]. Within our training set, we used a complete k-fold cross-validation process, completely re-recreating our training set each time samples were excluded [15]. However, because the ultimate test of a signature is its performance on completely independent samples, we also created a validation set with samples that were not included in the development of our signatures. These samples were procured at different centers, the tissue was handled differently after procurement, the microarray experiments were conducted in different laboratories, and the slides were read by different pathologists. Despite these potential sources of error, our classifier still maintained a 94% correct classification rate.

The ability to perform both cross-validation and independent validation allowed us to study the effects of different classification methods on the rate of correct classification. The two different classifier types, nearest centroid and Bayesian compound covariate predictor, had similar rates of misclassification/non-classification, but their utility may differ depending on the purpose of the user. If the goal is a dichotomous decision about molecular similarity between a given sample and the molecular signatures described in this study, the nearest centroid model is preferable. However, if avoiding false negatives is a priority and the user is willing to accept a number of non-classified samples, the Bayseisan compound covariate predictor may be preferable. An added advantage of the Bayesian compound covariate predictor is that the user is able to evaluate a numeric probability of a sample's membership in a given class.

Our gene signatures were resilient to changes decreasing signature size. Our ten gene signatures performed almost as well as our 50 gene signatures, demonstrating the robust nature of our analyses. Similar to the MammaPrint® signature platform that is being evaluated to determine the use of gene signatures in early stage breast cancer patients who may benefit from adjuvant chemotherapy, our signatures may potentially be used in a clinical setting [36]. We are considering a clinical trial to test the use of our algorithm in clinical practice, and the potential utilities are numerous. It may be used as a helpful adjunct to pathologists when the diagnosis are not obvious, it may improve the diagnostic accuracy of percutaneous renal biopsies (when the paucity of the tissue limits adequate evaluation of morphologic patterns), and it may help direct treatment options. For example, separating benign oncocytomas that may safely be observed from chromophobe tumors with malignant potential may be aided with the use of our signatures. Of most importance, our potential ability to achieve correct diagnosis in the majority of cases is intriguing and promising.

While the purpose of the analysis was to identify the transcripts most useful in differentiating different histologic subtypes, evaluation of the lists of transcripts revealed several interesting findings. First, the genes coding for some of the traditional immunohistochemical markers for differentiating tumors were found within the signatures. For example, in the 50 gene signature that differentiated clear/papillary from chromophobe/oncocytoma, the clear cell marker Vimentin was identified. In the 25 gene signature that differentiated chromophobe from oncocytoma, the chromophobe-specific marker Cytokeratin 7 was included [34]. Thorough evaluation of each of the genes in the signatures is outside the scope of this study, although we did note that several of the genes included had a known role in oncogenesis. For example, PAX-8 was listed as over-expressed in clear cell tumors relative to papillary. PAX-8 has been implicated in Wilm's tumors [37]. SCRN1, a marker of colorectal cancer, was also over-expressed in ccRCC relative to papillary [38]. Some of the genes in the signatures may be “observers” rather than “drivers”. For example, the over-expression of aquaporin 6 in chromophobe tumors relative to oncocytomas is unlikely to have any direct effect on tumor growth or invasion, but is nonetheless a good marker - this is the second study to show differential expression of this gene between chromophobe RCC and oncocytomas [39].

We acknowledge several limitations of this study. First, while the study includes the most common types of renal cortical tumors, not all histologic subtypes are represented in our model. For example, we excluded collecting duct RCC, clear cell RCC with papillary features, and tubulo-papillary RCC. We felt that most of these sub-types are either very rare or represent heterogeneous histologies. We also did not separate papillary tumors into type I and type II. These tumors may represent a continuum rather than separate entities, and papillary type II may include eosinophilic tumors of many origins [40]. We also did not performed analysis of normal renal parenchyma. Normal renal parenchyma can be readily distinguished from a solid renal tumor on H&E stain. Therefore, we focused on differentiating different types of RCC. Furthermore, while we validated the robustness of this signature by examining the performance of our signature in outside datasets, the true performance of the signature will have to be confirmed in a set of unclassified renal cortical neoplasms. Indeed, this may prove to be the most useful utility of these signatures. Finally, we did not incorporate stage and grade of the tumors into our algorithm as this information was not available for all studies.

While the lack of centralized pathology review may be considered as a limitation of our study, it is an inherent feature of this study that may also be its strength, since the samples analyzed were derived from multiple institutions in two countries. Our 94% accuracy when validated on external datasets is likely due to the use of data from multiple sources making our results more generalizable. The strong performance of our signature even in the non-clear histologic subtypes is best explained by the fact that we were not evaluating the genes that correlated with a histologic subtype defined by one pathologist, but rather the integration of molecular profiling with morphology as determined by multiple pathologists at various academic institutions.

We hope that our future studies will help address the current shortcomings in subclassification of renal cortical tumors, support the clinical utility of our algorithm, and move the field closer towards personalized medicine for patients with renal cortical neoplasms.

In summary, the use of meta-analytic techniques has facilitated the creation of signatures that have accurately differentiated renal cortical neoplasms. Sequential application of three signatures, according to an algorithm that utilized the natural differences in gene expression between tumor subtypes, correctly classified renal epithelial tumors from five institutions with 94% accuracy. Our algorithm may potentially be used as a adjunct for pathologists when the diagnoses are not obvious in order to improve the diagnostic accuracy of percutaneous renal biopsies and to help direct treatment options.

## Supporting Information

Table S1 **Gene Signature.** ccRCC - Clear Cell Renal Cell Carcinoma; pRCC - Papillary Renal Cell Carcinoma; chRCC - Chromophobe Renal Cell Carcinoma; ONCO – Oncocytoma.
(XLS)
